# Degradation and Removal of Acrylic/Alkyd Hybrid Paint Using Soybean Oil-Derived Methyl Esters as Bio-Based Solvents

**DOI:** 10.1021/acsomega.6c03913

**Published:** 2026-09-04

**Authors:** Elsy T. Vega-Lizama, Luis Díaz-Ballote

**Affiliations:** Department of Applied Physics, Center for Research and Advanced Studies, Campus Mérida, km 6 Carretera Antigua a Progreso, 97310 Cordemex, Mérida, Yucatán, Mexico

## Abstract

Acrylic/alkyd hybrid coatings effectively combine the superior adhesion and gloss of alkyd resins with the chemical and mechanical resilience of acrylic polymers. However, their removal typically necessitates the use of hazardous petroleum-derived solvents. This study evaluates the application of vegetable oil methyl esters (VOMEs)—synthesized via alkaline transesterification of soybean oil—as a biobased and efficient alternative for paint degradation and removal. Commercial acrylic/alkyd paint films were exposed to VOMEs for varying durations (15–120 min) and characterized using mass change analysis, Fourier transform infrared spectroscopy with attenuated total reflection (FTIR-ATR), thermogravimetric analysis (TGA), and elongation testing. Mass change analysis indicated a maximum film mass increase of 10.51% after 120 min, due to the sorption of VOMEs into the paint film. The TGA data revealed an additional mass loss between 150 and 260 °C in the treated films, attributed to the thermal release of sorbed VOMEs. Furthermore, the FTIR-ATR spectra demonstrated that this solvent uptake disrupted the cross-linking and aromatic structures of the paint film. Mechanical characterization confirmed significant structural weakening, as evidenced by a 97% reduction in time to failure. Practical assays on glass, carbon steel plates and cladding stone substrates achieved complete paint removal within 55 min. Thermogravimetric analysis confirmed that these biobased VOMEs remain thermally stable up to 150 °C, preventing premature thermal release during stripping operations and positioning them as promising, environmentally friendly alternatives to conventional stripping agents. Hansen solubility parameters and relative energy difference values indicate that the main VOMEs components drive the stripping action by weakening interchain forces, which promotes blistering, curling, and swelling and thereby facilitate coating removal.

## Graphical abstract

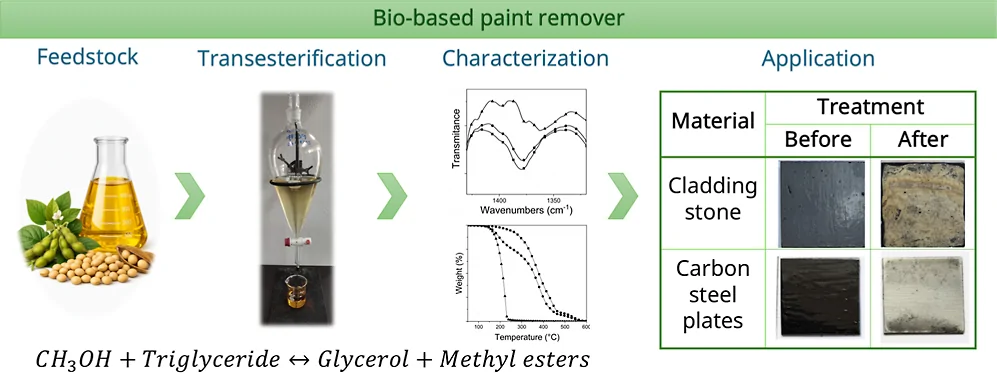

## 1 Introduction

The utilization of alkyd resins and acrylic polymers has expanded considerably in recent years because of their advantageous physicochemical properties in the formulation of paints and coatings.^5^ Alkyds exhibit robust thermal, chemical, and mechanical resistance, whereas acrylics offer enhanced flexibility and hardness and superior resistance to abrasion, alkalis, and moisture.^1^ Consequently, these polymers are widely employed as binders in surface coatings across diverse industrial sectors. Alkyd resins are synthetic polyesters formed by the high temperature polycondensation of polyols, fatty acids, and petrochemical anhydrides. While renewable oils and glycerol generally comprise 60% to 70% of the resin, phthalic anhydride remains an essential fossil-derived ingredient. This petrochemical component typically represents 26% of the structure and is notably not biodegradable. Therefore, the final resin retains a hybrid character that blends biosourced components with fossil-origin stability.^2–7^ Conversely, acrylic compounds are synthesized from fossil-based feedstocks and have been extensively applied in the production of paper, textiles, and high-performance coatings.^8^ Driven by the complementary attributes of these two polymer classes, the development of acrylic/alkyd hybrid coatings has gained significant momentum. These hybrids integrate the superior gloss and adhesion of alkyds with the chemical and physical resilience characteristics of acrylics, rendering them ideal for a broad spectrum of applications, including corrosion protection, construction materials, automotive components, wood preservation, and the restoration of artistic surfaces.^1–5^

Despite their versatility and widespread implementation, the removal of acrylic/alkyd hybrid paint remains heavily contingent upon petroleum-derived solvents. These include aromatic hydrocarbons such as xylene, ethylbenzene, and toluene, which are primary constituents of commercial reducers such as Reducer 73.^9^ Such solvents are characterized by high flammability, acute toxicity upon inhalation or dermal contact, and significant environmental persistence.^9^ In light of the global shift toward sustainable technologies, the development of effective, biobased, and nontoxic alternatives is imperative.

Various strategies have been proposed to mitigate these concerns. For instance, ionic liquids have been investigated as green solvents for stripping alkyd paint from wooden substrates.^10^ However, while they reduce adhesion, they often require prohibitively prolonged exposure times (up to 120 h). Other studies have explored nanostructured surfactant-based fluids for specialized applications, such as graffiti removal from sensitive surfaces such as marble.^11–15^ Nevertheless, these alternatives frequently encounter substantial limitations, including intricate preparation protocols, the need for high-pressure processing, and restricted compatibility with diverse substrates.

A critical gap persists in the current literature regarding the availability of versatile, sustainable solvents capable of effectively removing acrylic/alkyd hybrid paints from varied surfaces. Most extant research focuses on specific substrates or highly niche applications, thereby limiting the scalability and generalizability of the proposed solutions.^10,12,13^ VOMEs have emerged as promising candidates for addressing this gap. These biobased compounds are reported to be biodegradable, nontoxic, free of heavy metals, and exhibit high thermal stability (up to 150 °C) when synthesized via straightforward routes.^16,17^ Their inherent amphiphilic nature may facilitate favorable interactions with complex polymer matrices, suggesting their significant potential as efficient paint-stripping agents.

The present study investigated the degradation of acrylic/alkyd hybrid paints upon exposure to VOMEs to evaluate their efficacy as biobased solvent alternatives across varied material surfaces. Glass, carbon steel, and standard stone cladding were used as substrates to simulate typical industrial and structural finishing applications. To this end, the effects of VOMEs on film mass, structural composition (via FTIR–ATR), thermal stability (via TGA), and mechanical integrity (via elongation analysis) were systematically examined.

## 2 Materials and Methods

### 2.1 Preparation and Treatment of Paint Films

Acrylic/alkyd hybrid films were fabricated using a commercial high-gloss black acrylic enamel (Kem Fast Dry, Línea G69, Sherwin-Williams). The formulation consists of a chemically resistant acrylic resin matrix modified with alkyd resin with a total solids content of 57.86 ± 2% by weight (41.71 ± 2% by volume), a specific gravity of 1.196 ± 2% kg L^–1^, and a maximum volatile organic compound (VOC) content of 501.47 g L^–1^. This commercial paint utilizes carbon black as the primary pigment phase and is engineered for application on structural carbon steel and architectural substrates.^18^ The parent films were cast into silicone molds to produce specimen strips with precise dimensions: 0.40 ± 0.05 mm in thickness, 25 ± 0.10 mm in width, and 75 ± 0.1 mm in length. After 6 days of drying (at 25 °C and 1 atm), the films were removed from the mold and placed on a Teflon plate until the drying time was complete. Following a 15 day ambient drying period (See Section 2.3), parent paint films were cut into test films (25 ± 0.1 mm long, 3.60 ± 0.10 mm width, and 0.40 ± 0.05 mm thick). Test films were positioned inside a concave watch glass. A single VOMEs drop (0.024 ± 0.001 g) —whose average mass was precalibrated gravimetrically on an analytical balance—was deposited precisely on the geometric center of the film; the concave profile of the glass effectively maintained the drop in position, avoiding runoff. The drop remained in contact with the films for intervals of 15, 30, 45, 60, or 120 min. Only the drying step was performed using the parent films; the remaining experiments were conducted using the test films and employing a single-drop treatment with VOMEs. Residual VOMEs were subsequently removed using Whatman No. 1 ash-free filter paper. Untreated paint films stored under identical ambient conditions served as the baseline control samples to evaluate the structural, chemical, and mechanical modifications induced exclusively by the biobased ester exposure. All treatments and characterization procedures were conducted in triplicate to ensure experimental reproducibility. Samples were designated using the prefix “AA” (acrylic/alkyd), followed by a numerical value representing the treatment duration in minutes (e.g., AA15 to AA120).

### 2.2 Synthesis and Characterization of VOMEs

Vegetable oil methyl esters (VOMEs) were synthesized via homogeneous catalytic transesterification of soybean oil, as briefly described elsewhere.^19^ Following transesterification, the reaction mixture was allowed to settle overnight to separate and remove the glycerol phase. The crude VOMEs were subsequently washed with distilled water for approximately ten successive cycles until a clear aqueous effluent was obtained. This intensive washing process exploits the high-water solubility of ionic catalyst residues (such as potassium), effectively partitioning them into the discarded aqueous phase to yield a purified hydrophobic ester matrix. The resulting VOMEs were dried at 110 °C for 4 h, allowed to cool, and then utilized for the paint degradation experiments. The acid value was determined to be 0.9 mg KOH g^–1^ following the ASTM D974-22 standard, while gas chromatography (AOCS Ce 1h-05) was employed to verify the qualitative fatty acid profile of the VOMEs. Additionally, the kinematic viscosity and density were also determined through the ASTM D445 and ASTM D1298 respectively. Thermogravimetric analysis (TGA) was utilized to assess the purity (total amount of esters)^19,20^ and thermal stability of the bulk VOMEs based on their characteristic decomposition temperature range and final ash content, confirming successful conversion. The final product was stored in amber glass containers under 25 C° and 1 atm (standard laboratory conditions).

### 2.3 Evaluation of Mass Changes

Two distinct mass measurements were performed: (a) the drying time was established by weighing fresh parent films daily until mass stabilization was achieved; (b) to evaluate degradation, the percentage of mass change of the test films, following VOMEs exposure was calculated using eq 1:

1 $$\Delta M=\left(\frac{M_{2}-M_{1}}{M_{1}}\right)\times 100$$

where *M*_1_ and *M*_2_ represent the initial and final masses of the films, respectively. In the latter case, these trials were conducted under standard ambient conditions (25 °C and 1 atm), the potential mass loss of the solvent via evaporation over the measuring period was considered negligible. This assumption is supported by two factors: (a) VOMEs are not volatile up to 150 °C, and (b) soybean oil fatty acid methyl esters exhibit characteristically low vapor pressure at room temperature, on the order of 10^–4^ Pa^22^.

### 2.4 Thermogravimetric Analysis

TGA was conducted using a Discovery Series thermobalance (TA Instruments). Samples (10.0 ± 0.5 mg) were heated from 50 to 600 °C at a constant rate of 10 °C/min under a dry air flow of 25 mL/min. Measurements were performed in triplicate for both untreated and VOMEs-exposed samples.

### 2.5 FTIR–ATR Spectroscopy

Fourier transform infrared spectra were acquired using a Nicolet iS5 spectrometer (Thermo Scientific) equipped with a diamond ATR accessory (iD7 module, single bounce). Spectra were collected over the 4000–400 cm^–1^ range with a resolution of 16 scans per sample. Interference from atmospheric CO_2_ and humidity was digitally suppressed. Each experimental condition, including the control samples, was analyzed in triplicate.

### 2.6 Elongation Testing

Mechanical elongation assays were conducted at room temperature. Each film was suspended vertically alongside a Mitutoyo steel rule, 30”/750 mm, and a constant load of 20.8 g was applied (see Supporting Information, Figure S1). This load was selected after preliminary weight trials to ensure a stable and controllable deformation rate. To guarantee high precision and eliminate human reading errors, the elongation process was continuously recorded using a digital video camera tracking both the scale and an adjacent chronometer simultaneously, allowing for subsequent frame-by-frame synchronized analysis of time and displacement data.

### 2.7 VOME Stripping Efficiency on Typical Real Surfaces

To evaluate the stripping efficiency of the VOMEs across diverse industrial substrates, comparative assays were performed on three surfaces: nonporous glass slides, carbon steel plates (3.0 × 3.0 cm), and cladding stone (10 × 10 cm). Before coating, the surface of carbon steel plates was standardized with 600 grit silicon carbide paper, while the cladding stone was utilized in its as-received condition to preserve its inherent surface rugosity. All substrates were coated with three layers of acrylic/alkyd enamel according to manufacturer specifications and left to dry under controlled laboratory temperature of 25 °C for 5 days. The stripping evaluation was conducted via static contact trials, where the coated side of each specimen was exposed to 10 mL of VOMEs in a closed vessel (Petri dish). Exposure intervals were optimized for each substrate based on their adhesion characteristics: glass slides were analyzed from 5 to 30 min, carbon steel from 5 to 35 min, and cladding stone from 15 to 55 min. Following each interval, the softened paint matrix was mechanically removed using a standardized rubbing protocol (10 linear strokes under constant pressure with a lint-free cotton cloth), adapted from ASTM D4828 and ASTM D3450 standards. The percentage of paint loss was subsequently quantified using the mass change formula described above.

## 3 Results and Discussion

### 3.1 Physicochemical Characterization of VOMEs

The VOMEs synthesized in this study exhibited a high macro purity of 98.5%, as determined by thermogravimetric analysis (TGA).^19,20^ The postsynthesis drying stage (110 °C for 4 h) ensured the complete removal of volatile compounds, including methanol (boiling point ≫ 64.7 °C) and moisture. The remaining 1.5% of the mass balance is attributed to the presence of mono-, di-, and triglycerides remaining from the transesterification process used to produce the VOMEs.^21^ These components do not compromise the solvent power of the VOMEs (See Section 3.6). Furthermore, the VOMEs displayed a relative density of 0.87, a kinematic viscosity of 4 mm^2^ s^–1^ (at 40 °C), and an acid value of 0.9 mg KOH g^–1^ These properties are consistent with previously reported values.^19^ Hybrid acrylic/alkyd paint combines an acrylic matrix, a highly polar thermoplastic polymer, with an alkyd matrix, a thermoset polymer that cures through oxidative cross-linking of its unsaturation. In this system, VOMEs function as biobased remover because: the ester functional group enhances the chemical affinity with the acrylic chains, improving their solvation capacity; saturated and monounsaturated chains help maintain a flexible structure, facilitating penetration into the hybrid molecular network and weaken intermolecular interactions,^22^ meanwhile polyunsaturated fractions, which represent approximately 57% of the raw material (Table 1), lower the melting point and help keep the remover in a liquid state with low viscosity.^23^ It has been found that a viscosity of around 4–5 mm^2^ s^–1^ at 40 °C possessed by VOMEs gives an excellent capacity for penetration into the micropores of the paint layer.^24^

**Table 1:** Soybean Fatty Acid Profile Obtained via Gas Chromatography

saturation	fatty acid	weight percentage
polyunsaturated	9,12-octadecadienoic acid/linoleic acid	50
	9,12, 15-Octadecatrienoic acid/linolenic acid	7
monounsaturated	9-octadecenoic acid/oleic acid	20
saturated	hexadecanoic acid/Palmitic acid	11
	stearic acid	3

### 3.2 Mass Change

The drying kinetics of the acrylic/alkyd parent films (Figure 1a) resulted in a 50.56% mass reduction over a 12 day period because of solvent evaporation, reaching stabilization at 1.26 ± 0.01 g. This mass loss correlates precisely with the volatile organic compound (VOC) content of 501.469 g L^–1^ specified by the manufacturer. Consequently, a 15 day drying period was established as a baseline prior to subsequent testing. During drying, the maximum standard deviation in mass change was ±0.006 g, while the standard deviation for percentage mass change ranged from ±0.261% to ±0.549%.

**Figure 1:**
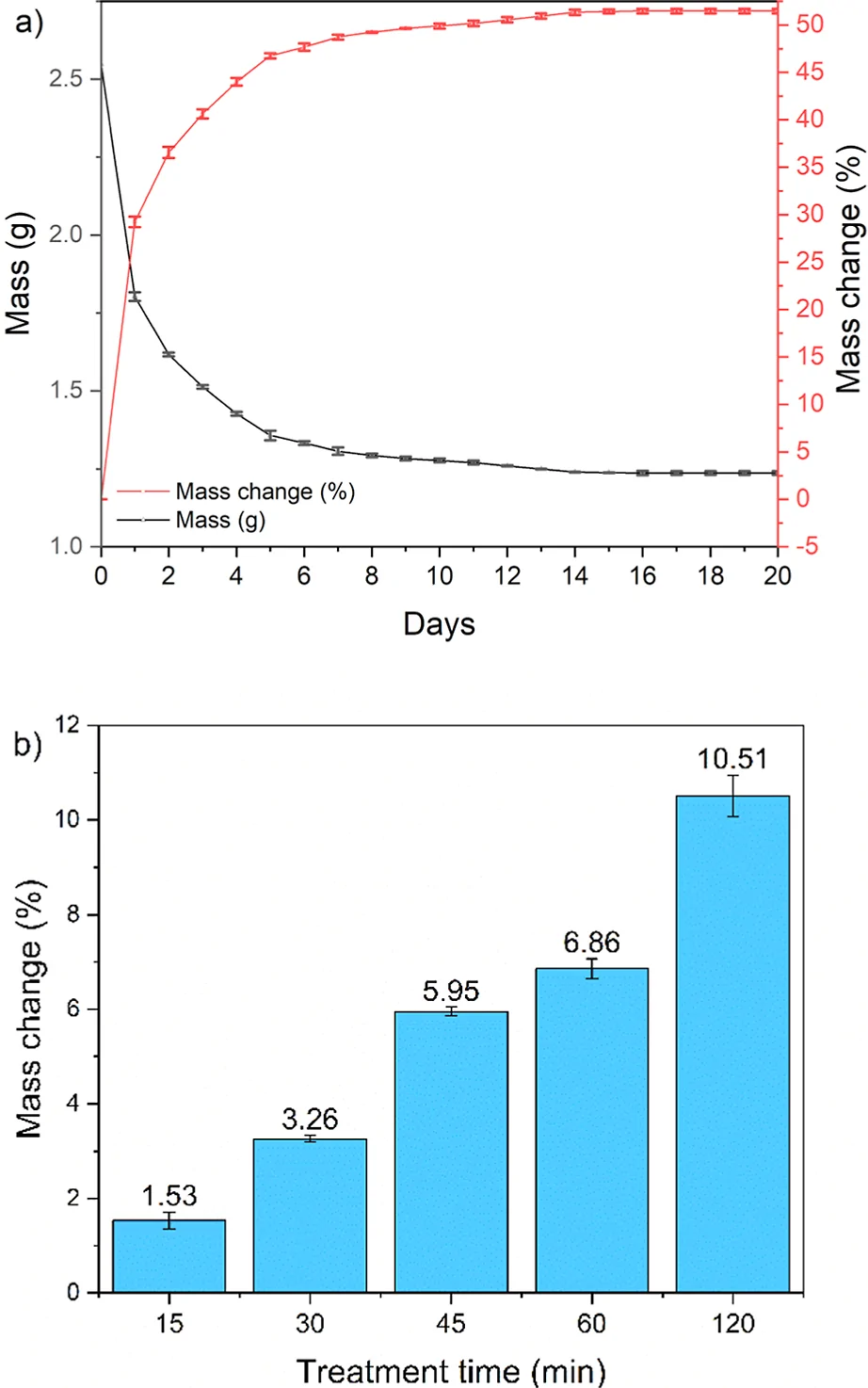
(a) Nonforced ambient temperature drying curves of acrylic/alkyd hybrid paint films showing absolute mass (g) and weight change (%) due to solvent evaporation; (b) Mass change percentage of the films after treatment with WOMEs at different contact times.

The test films weighed 0.069 ± 0.003 g prior to treatment with VOMEs. Upon exposure to VOMEs, the mass gain of the films progressively and significantly increased with respect to the contact time. A maximum increase of 10.51 ± 0.32% was recorded after 120 min (Figure 1b). This swelling behavior provides macroscopic evidence that VOMEs successfully penetrated the paint matrix through physical sorption mechanisms. Such uptake is likely driven by two coupled mechanisms: first, methyl esters have a structural affinity with the alkyd fatty chains allowing the solvent to penetrate between polymer chains and disrupt intermolecular forces;^25^ second, although they do not rapidly cleave the polymer backbone at room temperature, they induce softening of ester-rich regions in the alkyd network, progressively converting the cured coating into a weakened, removable mass.^24^ Therefore, mass change analysis provides primary evidence of effective molecular-level interactions between the biosolvent and the hybrid coating.

### 3.3 Thermogravimetric Analysis

The thermograms revealed three distinct stages of mass loss during the thermal decomposition (Figure 2). The first region designated ZONE 1 (150–260 °C) corresponds to the volatilization of the sorbed VOMEs, which consist of fatty acid methyl esters with characteristic chain lengths spanning between 17 and 23 carbon atoms.^26,30^ This temperature assignment is thoroughly consistent with the distillation profile of refined soybean biodiesel reported by Chand et al.^27^ and is validated by our baseline measurement of pure VOMEs (yellow curve), which exhibits a complete and sharp weight loss within this exact thermal window.

**Figure 2:**
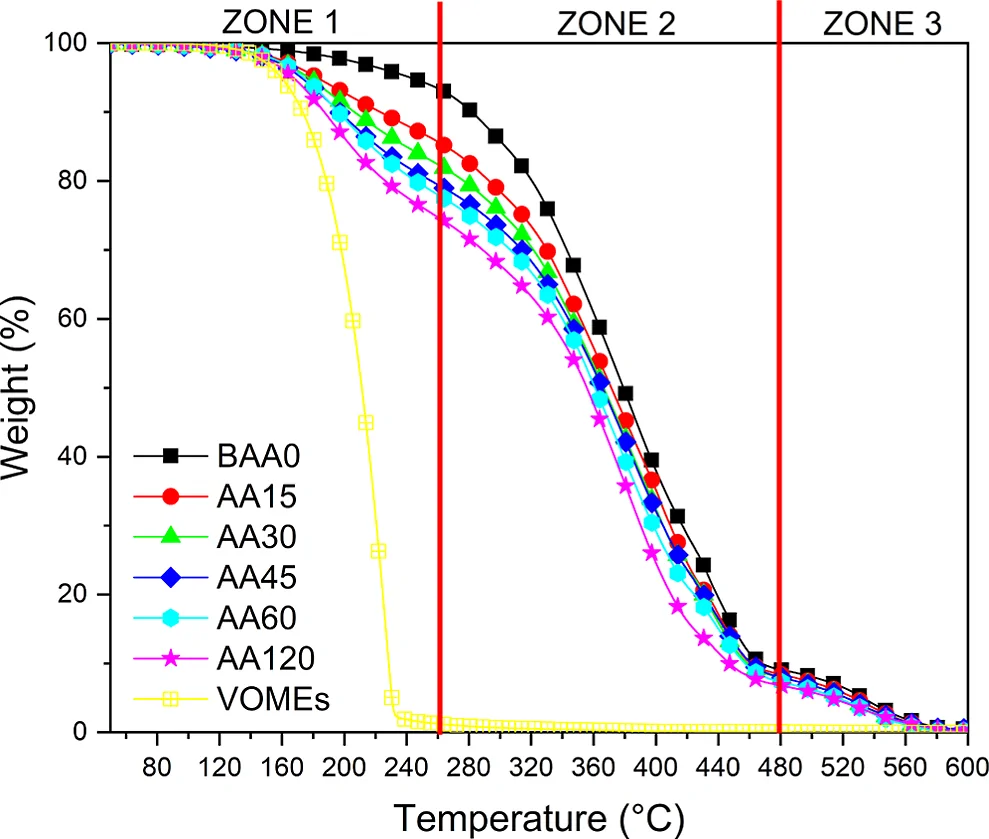
Typical TGA thermograms of films with treatment from AA15 to AA120 (where the number corresponds to the contact time in minutes), without treatment (BAA0) and with VOMEs.

While untreated samples (BAA0) exhibited a negligible loss of 5.0% (value taken at 260 °C) in this zone (attribute to inherent volatile additives), the samples exposed to VOMEs demonstrated a progressive displacement toward lower weight percentage that peaked at a 25.2% mass loss for the AA120 sample. Subtracting the 5.0% baseline additives loss from the total 25.2% TGA drop yields a net sorbed VOMEs mass loss of w_VOMEs,TGA_ = 20.2%. Although this local TGA concentration appears higher than the macro-gravimetric solvent uptake recorded for the whole film (10.51 ± 0.32%, Figure 1b), this numerical difference is governed by a spatial scale disparity in sampling. To preserve unexposed peripheral regions for mechanical jaw-clamping during subsequent elongation tests, each VOMEs biosolvent droplet was deposited precisely at the geometric center of the paint film (25 mm × 3.6 mm, total film area, *A*_total_ = 90 mm^2^). Upon application, the droplet spread along the film length, forming a central wet footprint with a shape close to a rectangle of 13.8 mm long × 3.6 mm width and *A*_rect_ = 49.7 mm^2^. While microscale TGA specimens were excised exclusively from this central wet footprint to capture the intensive local saturation thresholds of the coating, macro-gravimetry measured the mass change across the entire film. The relationship linking both analytical techniques is thus governed by spatial normalization (eq 2):

2 $$\%\Delta m_{global,predicted}=w_{VOMEs,TGA}\times f_{area}$$

where *f*_area_ = *A*_rect_/*A*_total_. Applying this spatial normalization factor to the sample exposed for 120 min (*w*_VOME,TGA_ = 20.2%) predicts a global mass increase of 11.15%, which shows good agreement with the experimental gravimetric sorption of 10.51% (an absolute deviation of only 0.64%). Both techniques consistently confirm the same physical trend: a significant, time-dependent uptake of VOMEs into the hybrid paint structure. representing the physical accumulation and stable retention of the biobased solvent within the inner free volume of the hybrid coating. The experimental TGA curves verified the high thermal stability of the bulk VOMEs up to 150 °C, confirming that no premature thermal release or volatilization occurs under ambient operational conditions. This extended stability prolongs fluid contact and drives the physical swelling of the matrix, especially in acrylic domains, which generates internal stress, wrinkling, and loss of adhesion to the substrate.^28^

In ZONE 2 (260–480 °C), pronounced mass loss reflects the thermal decomposition of the cross-linked acrylic/alkyd network. As the initial VOME exposure time increased, the onset of this main decomposition step shifted toward lower temperatures and the degradation rate accelerated. This behavior can be rationalized by two coupled phenomena supported by the thermal cracking principles of fatty esters described by Luo et al.:^29^ first, the infiltrated solvent significantly reduces the cohesive energy density of the polymer network, acting as an internal plasticizer that enhances chain mobility; and second, at temperatures exceeding 300 °C, the entrapped aliphatic chains undergo substructural thermal cracking and decarboxylation pathways. supporting the hypothesis that VOMEs infiltration effectively weakens polymer cross-linking.

Finally, in ZONE 3 (above 480 °C) a consistent residual mass of approximately 6% remained in all samples above 480 °C, which is attributed to thermally stable inorganic filler, pigments, additives such as carbon black and CaCO_3_, as established in the literature.^30–32^ Thus, the TGA results provide evidence that mirrors the gravimetric mass change findings, offering a comprehensive insight into the degradation mechanisms.

### 3.4 FTIR–ATR Spectroscopy

The FTIR spectra corroborated the occurrence of chemical interactions between the VOMEs and the paint matrix. Bands associated with methylene (−CH_2_−) and ester groups (e.g., 2925, 2852, and 1192 cm^–1^) intensified in direct proportion to exposure time (Figure 3), reflecting the increased concentration of VOMEs within the film. Moreover, the bands at 1379 cm^–1^ and 1257 cm^–1^, associated with the CH_2_/NCO deformation of the cross-linker (Figure 3b), as well as aromatic C–H vibrations at 1598, 1578, and 740 cm^–1^, decreased significantly.^33^ This spectral evolution confirms two concurrent phenomena: the deep penetration of VOMEs into the film and the subsequent disruption of the polymer network, particularly within the cross-linked and aromatic domains. These molecular-level findings align seamlessly with the TGA and mass gain data.

**Figure 3:**
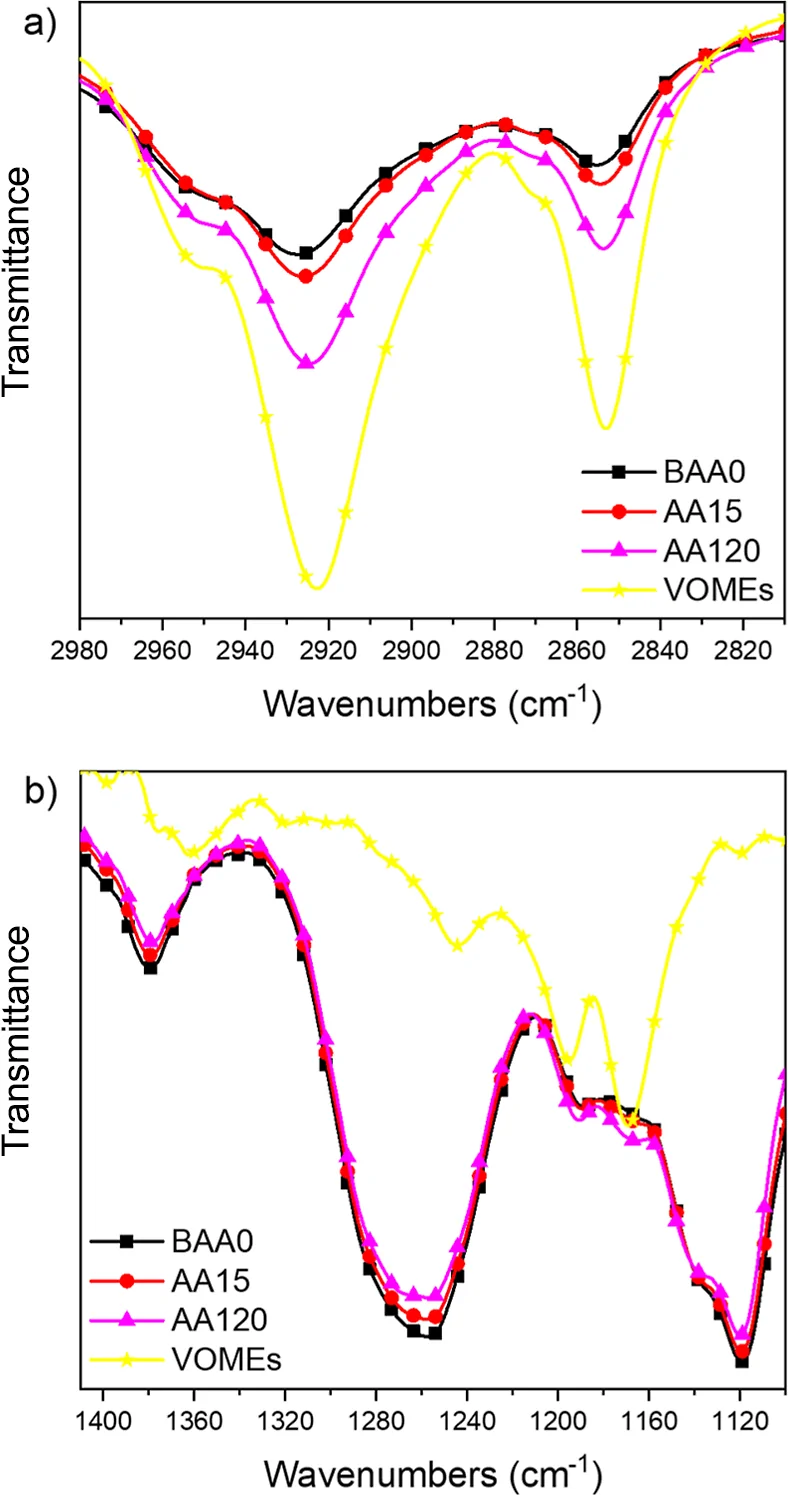
Typical bands for VOMEs, the blank sample, and samples exposed for 15 and 120 min (a) Bands at 2925–2850 cm^–1^ (C–H stretching); (b) Bands at 1379 cm^–1^ related to the CH_2_/NCO deformation of the cross-linker; increased bands at 1192 and 1167 cm^–1^ related to ester stretching.

### 3.5 Mechanical Degradation and Elongation Behavior

As illustrated in Figure 4, mechanical testing revealed that prolonged exposure to VOMEs severely compromised the elongation capacity and structural integrity of the hybrid films. After 15 min of exposure, the films reached an elongation of 44 mm prior to rupture; however, samples exposed for 120 min fractured at only 24 mm. Concurrently, the elongation velocity increased by an order of magnitude, from 0.005 mm/s (15 min) to 0.05 mm/s (120 min), indicating a drastic acceleration of deformation under constant loading.

**Figure 4:**
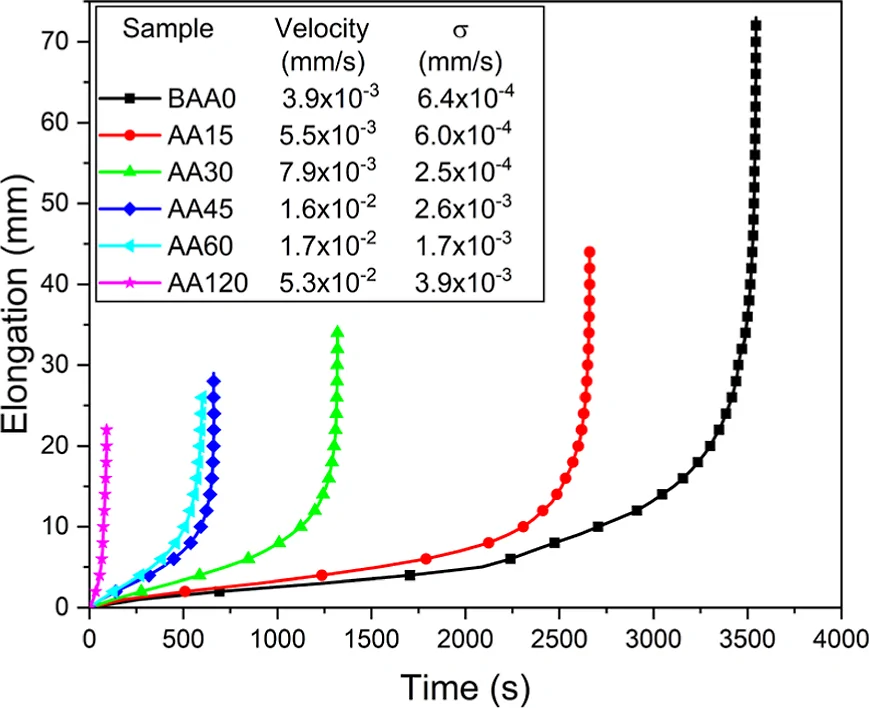
Elongation analysis of the average behavior, elongation velocity, and standard deviation of treated and untreated films.

The fracture time decreased precipitously from 3545 s in the untreated films to a mere 92 s after 120 min of exposure, representing a 97% reduction in time-to-failure. Consequently, these results provide compelling mechanical evidence that VOMEs disrupt the polymeric network, rendering the films significantly more brittle and susceptible to rapid failure under tensile stress. This mechanical failure profile is entirely consistent with the structural and chemical alterations identified via FTIR-ATR and TGA.

### 3.6 Thermodynamic Framework

Hansen solubility parameters (HSPs) extend Hildebrand solubility theory by accounting for specific noncovalent molecular interactions beyond simple dispersion forces.^34^ The total cohesive energy density of a given substance (δ_T_) is partitioned into three additive interaction components: dispersion forces (δ_D_), polar forces (δ_P_), and hydrogen bonding (δ_H_), related through eq 3:

3 $${\delta_{T}}^{2}={\delta_{D}}^{2}+{\delta_{P}}^{2}+{\delta_{H}}^{2}$$

In three-dimensional HSP space (δ_D_, δ_P_, δ_H_), a polymer’s solubility domain is represented as a sphere centered at its specific parameter coordinates, bound by an interaction radius (*R*_0_). The thermodynamic affinity between a solvent and a solute is quantified by the solubility distance (*R*_a_), where a weighting factor of 4 is applied to the dispersion term to yield a spherical solubility domain (eq 4):

4 $$R_{a}={\left[4{\left(\delta_{D,solvent}-\delta_{D,solute}\right)}^{2}+{\left(\delta_{P,solvent}-\delta_{P,solute}\right)}^{2}+{\left(\delta_{H,solvent}-\delta_{H,solute}\right)}^{2}\right]}^{1/2}$$

Relative solvency behavior is governed by the relative energy difference (RED = *R*_a_/*R*_0_).^34^ A RED <1.0 indicates a thermodynamically favorable solvent lying within the solubility sphere; a RED = 1.0 defines the boundary limit; and a RED >1.0 denotes a nonsolvent.^34^

To apply this thermodynamic framework to the biosolvent system, Acryloid B-44 was selected as a model solute representing the acrylic polymer matrix in the hybrid coating.^34^ Gas chromatography analysis of soybean oil established that linoleic acid (∼50%) and oleic acid (∼20%) constitute the predominant fatty acid chains. Following transesterification, thermogravimetric analysis confirmed a main conversion yield of 98.5% for VOMEs. The remaining 1.5% mass balance is attributed to unreacted residual acylglycerol species,^21^ specifically mono-, di-, and triacylglycerols (MAG, DAG, and TAG). Dynamic thermogravimetric decomposition of partial and full glycerides occurs within a range of 290–500 °C, confirming their retention within the nonvolatile thermal residue quantified by TGA.^21^

Table 2 summarizes the HSP components (δ_D_, δ_P_, δ_H_), calculated solubility distances (*R*_a_), and RED metrics for Acryloid B-44 (*R*_0_ = 10.5 MPa^1/2^) in contact with both active methyl esters and residual acylglycerol derivatives.

**Table 2:** Hansen Solubility Parameters, Solubility Distance, and Relative Energy Difference for Acryloid B-44 in Two Different Esters and Residual Acylglycerol Species

component/system	δ_D_ (MPa^1/2^)	δ_P_ (MPa^1/2^)	δ_H_ (MPa^1/2^)	*R*_a_ (MPa^1/2^)	RED	behavior
acryloid B-44 (Solute)^34^	19.40	11.20	4.40	—	—	target polymer
glyceryl monolinoleate (MAG)^35^	16.83	3.18	11.59	11.03	1.14	nonsolvent
glyceryl dilinoleate (DAG)^35^	16.49	1.72	7.30	11.50	1.09	nonsolvent
glyceryl trilinoleate (TAG)^35^	16.36	1.59	4.76	11.38	1.08	nonsolvent
methyl oleate (C18:1^38^	16.30	2.40	3.20	10.83	1.03	nonsolvent
methyl linoleate (C18:2)^36^	16.60	2.40	3.60	10.46	0.99	favorable/borderline

Substituting the coordinates of methyl linoleate into eq 3 yields an *R*_a_ of 10.46 MPa^1/2^. Dividing this distance by the interaction radius of Acryloid B-44 (*R*_0_ = 10.5 MPa^1/2^) produces a RED value of 0.996. Because RED is marginally below unity, methyl linoleate resides precisely on the thermodynamic threshold of solvency. In contrast, methyl oleate exhibits an *R*_a_ of 10.83 MPa^1/2^ and a RED of 1.03, placing it just outside the favorable solubility domain. When evaluating the acylglycerol species, glyceryl monolinoleate, dilinoleate, and trilinoleate yield RED values of 1.14, 1.09, and 1.08, respectively.

These quantitative metrics provide fundamental insights into the removal mechanism of the hybrid coating. Although mono- and diacylglycerols possess amphiphilic structures capable of displaying interfacial surfactant behavior, their RED values greater than 1.08 demonstrate a lack of thermodynamic affinity for the bulk acrylic resin.^35^ Furthermore, the bulky glycerol backbone in MAG, DAG, and TAG imparts significant steric hindrance and elevated molar volume, imposing kinetic diffusion barriers that hinder Fickian transport into the narrow intermolecular free volume of the polymer network. Consequently, the 1.5% residual acylglycerol fraction does not act as a primary solvency driver nor as an effective internal plasticizer.

Instead, the overall stripping efficacy is dictated by methyl linoleate, which constitutes ∼50% of the total biosolvent mixture. Operating at a borderline thermodynamic condition (RED = 0.996), linear methyl ester molecules penetrate the hybrid matrix. Rather than causing immediate, isotropic dissolution, methyl linoleate solvates localized acrylic segments and weakens interchain cohesive forces. This absorption increases segmental chain mobility and generates differential swelling pressure near the substrate interface. As internal mechanical stresses accumulate, the paint layer undergoes macroscopic blistering, curling, and structural weakening, ultimately enabling efficient coating removal.

### 3.7 Practical Efficacy: Paint Removal on Glass Substrates

To evaluate the practical viability of VOMEs, comparative removal assays were performed on glass slides, carbon steel plates, and cladding stone. It can be seen in Figure 5 that stripping kinetics are influenced by the surface adhesion mechanism. Glass, acting as a nonporous baseline with minimal interlocking, exhibited the most rapid kinetics: 54.6% removal at 5 min, reaching 100% detachment by 30 min (Figure 6). Similarly, carbon steel plates, despite the mechanical interlocking from abrasive finishing, achieved 77.2% removal at 15 min and 100% at 35 min (Figure 7). In contrast, the high porosity and rugosity of cladding stone facilitate penetration of paint into pores, cavities, and other surface flaws, creating a mechanical anchor that hinders removal. Consequently, stripping on cladding stone requires longer exposure, starting at 7.1% removal at 15 min and reaching a maximal degradation plateau of 93.8% at 55 min (Figure 8). These results confirm that while VOME-induced matrix swelling is consistent, the stripping rate is constrained by the mechanical interlocking strength of the substrate; nevertheless, all surfaces converge toward a maximal degradation threshold, validating the robust stripping efficiency of VOMEs on complex industrial surfaces (Figure 5).

**Figure 5:**
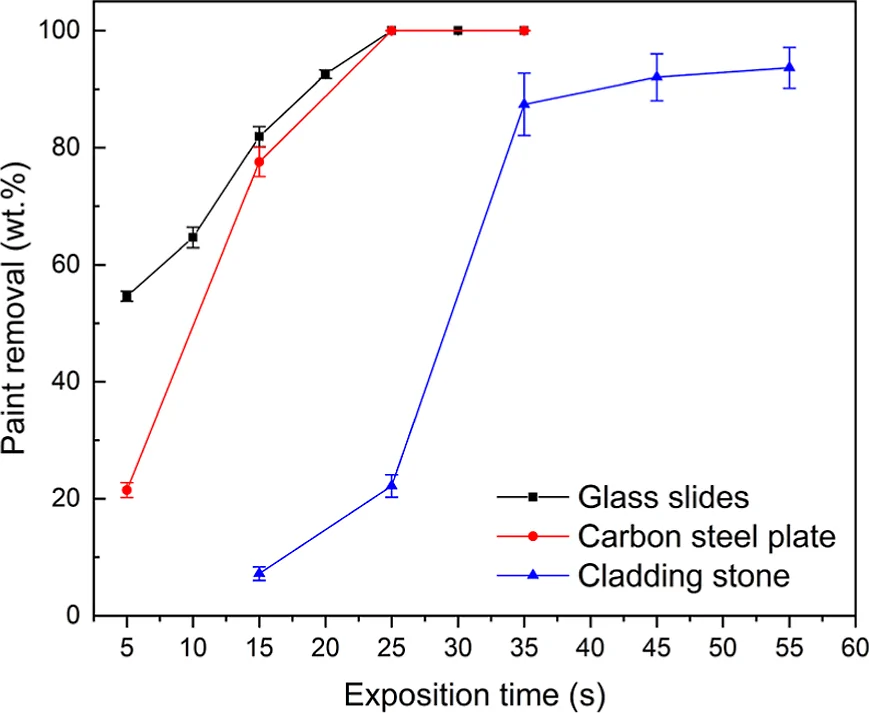
Comparative weight percentage of paint removed from glass, carbon steel, and cladding stone substrates after exposure to VOMEs.

**Figure 6:**
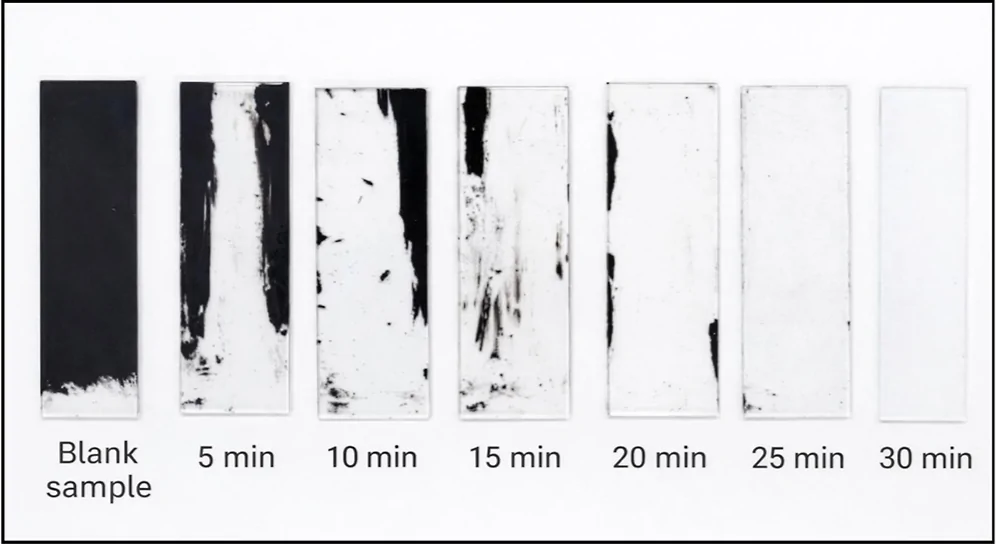
Visual progression of paint stripping on nonporous glass slides following VOMEs exposure over a 30 min period.

**Figure 7:**
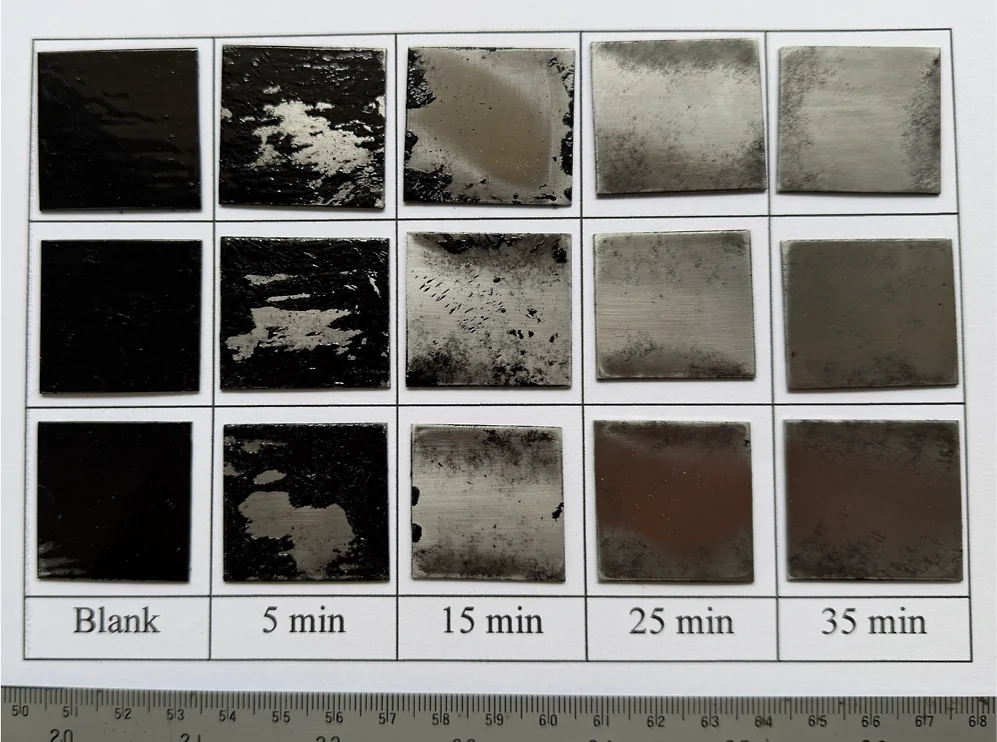
Visual progression of paint stripping on abrasive-finished carbon steel plates following VOMEs exposure over a 35 min period.

**Figure 8:**
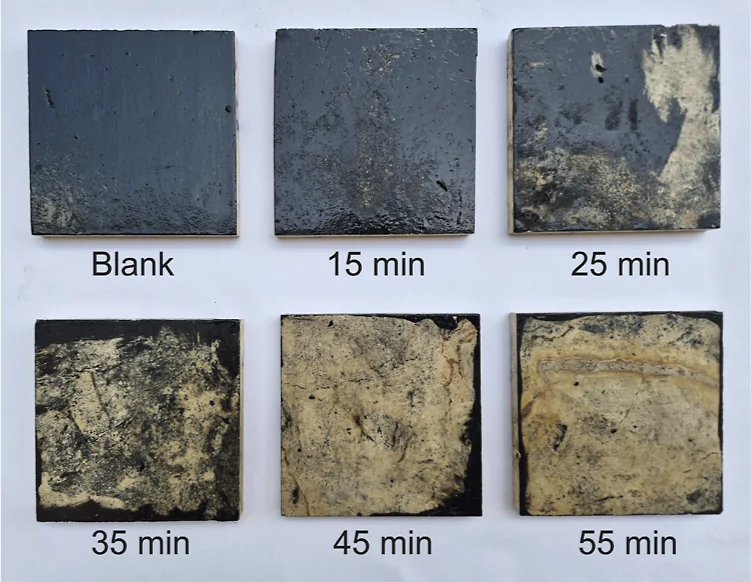
Visual progression of paint stripping on porous cladding stone surface following VOMEs application over a 55 min period.

## 4 Conclusions

This study demonstrates that VOMEs have significant potential as high-performance green solvents for the degradation and removal of acrylic/alkyd hybrid coatings. The experimental evidence confirms that VOMEs induce progressive and systematic structural alterations in paint films, with degradation kinetics being directly proportional to exposure time.

The robustness of this conclusion is supported by a multianalytical approach.

- Sorption and diffusion: A maximum mass increase of 10.51% indicates that VOMEs effectively penetrate the polymer matrix via sorption mechanisms.
- Structural disruption: FTIR–ATR and TGA analyses provide molecular and thermal confirmation of chemical interactions that disrupt the cross-linking and aromatic domains of the hybrid network.
- Mechanical failure: The 97% reduction in time-to-failure and the loss of elastic properties corroborate that solvent-induced swelling leads to critical structural weakening.
- Practical efficacy: The achievement of 100% paint removal on glass and carbon steel plates substrates within 30 and 35 min, respectively, validates the viability of VOMEs as a rapid and efficient alternative to conventional stripping agents.
- Hansen solubility parameters and relative energy difference values further support the foregoing, indicating that the main components of VOME drive the stripping process by weakening interchain forces and promoting blistering, curling, and swelling, thereby facilitating the removal of the coating

.From an ecological standpoint, compared with hazardous petroleum-derived solvents, VOMEs offer a superior profile, as they are biobased, nontoxic, and synthesized through low-energy processes. Consequently, their implementation aligns with global green chemistry initiatives to replace volatile and toxic compounds in surface cleaning and maintenance. In summary, this research establishes VOMEs as a biobased solvent and technically feasible solution for the removal of complex hybrid coatings. Future investigations should explore the performance of these biosolvents on a broader diversity of substrates and multilayer coating systems to further validate their industrial scalability.

## Supplementary Material



## Notes

Figure S1. Diagram of the setup for elongation testing (PDF)

## Abbreviations

VOMEs: vegetable oil methyl esters
AA: acrylic/alkyd
FTIR-ATR: Fourier transform infrared spectroscopy with attenuated total reflection
TGA: thermogravimetric analysis
VOC: volatile organic compounds
BAA0: blank samples.
