# Supplementary material for: Degradation and Removal of Acrylic/Alkyd Hybrid Paint Using Soybean Oil-Derived Methyl Esters as Bio-Based Solvents
Source: ACS Omega. 2026 Sep 4;11(37):55391–9. doi: 10.1021/acsomega.6c03913 (PMC13613430; doi:10.1021/acsomega.6c03913)
Supplement: Supplementary file 1 [file ao6c03913_si_001.pdf]

Degradation and Removal of Alkyd/acrylic Hybrid Paint using Soybean Oil-Derived Methyl Esters as Bio-Based Solvents

*Elsy T. Vega-Lizama and Luis Díaz-Ballote**

Department of Applied Physics, Center for Research and Advanced Studies, Campus Mérida, km 6 Carretera Antigua a Progreso, 97310, Cordemex, Mérida, Yucatán, México.

SUPPORTIN INFORMATION

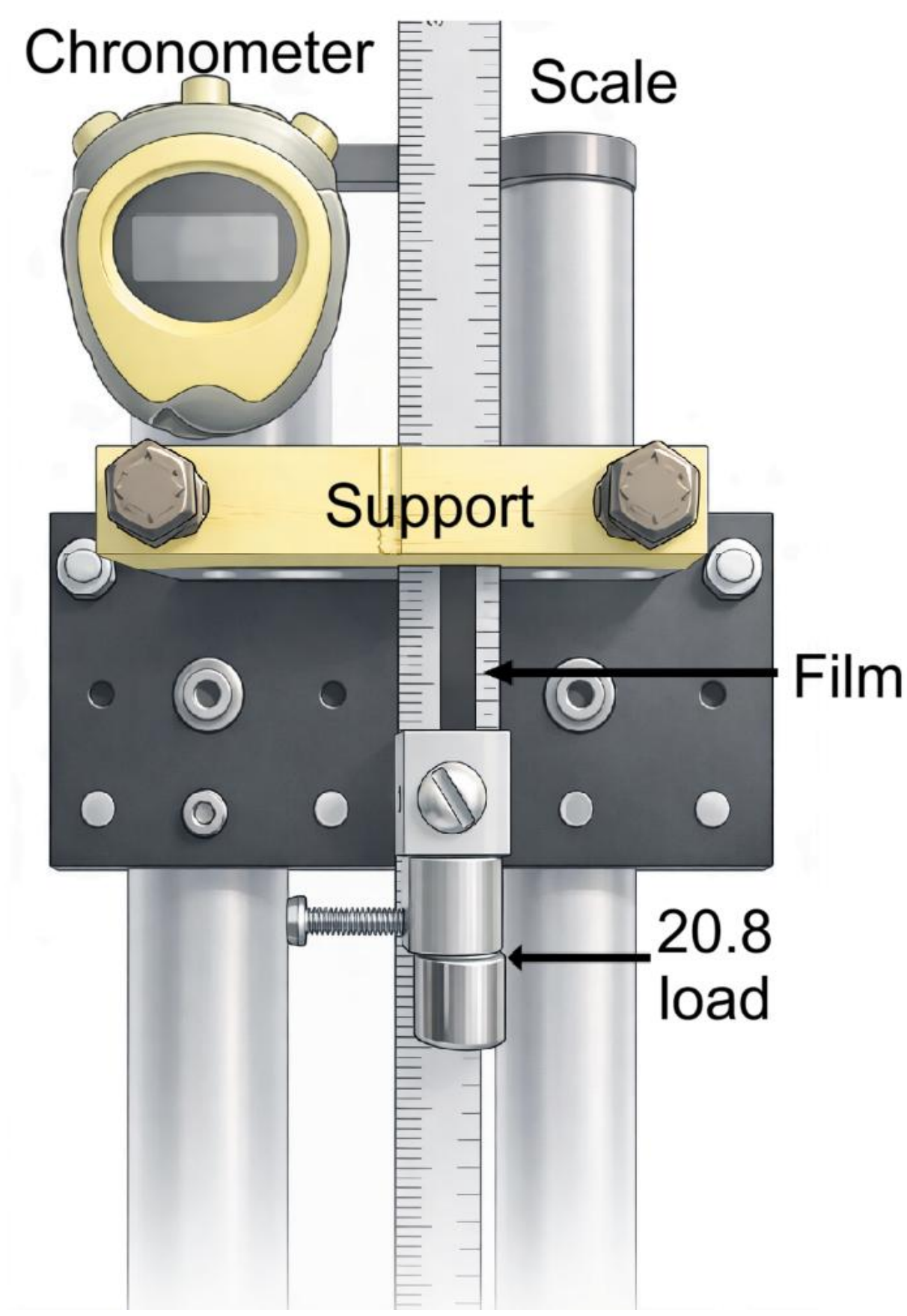

Figure S1. Diagram of the setup for elongation testing.
